# Standardization of Inpatient Insomnia Medications as a Potential Strategy to Reduce Outpatient Benzodiazepine Receptor Agonist Prescriptions

**DOI:** 10.7759/cureus.88952

**Published:** 2025-07-29

**Authors:** Asahi Omoto, Yuuka Shibata, Emiko Mashida, Erika Shigita, Tadakazu Shinmoto, Hiroaki Matsuo

**Affiliations:** 1 Department of Pharmaceutical Services, Hiroshima University Hospital, Hiroshima, JPN

**Keywords:** benzodiazepine, clinical pathway, hypnotic agent, insomnia, older adults

## Abstract

Introduction

Insomnia is a common clinical complaint, often presenting with symptoms such as difficulty initiating sleep, frequent nighttime awakenings, early morning awakenings, or non-restorative sleep. These disturbances are frequently associated with a range of psychiatric and physical conditions and may contribute to reduced quality of life and broader societal burden. Although benzodiazepines (BZ) are commonly used to manage inpatient insomnia, their use, especially in older adults, has been associated with adverse outcomes such as cognitive impairment, falls, and fractures. In an effort to promote safer prescribing practices, Hiroshima University Hospital revised its inpatient formulary and clinical pathway guidance in November 2021 to discourage the initiation of new BZRA prescriptions during hospitalization.

Methods

We conducted a retrospective review of medical records to analyze hypnotic prescriptions in outpatients, using descriptive statistical methods. This analysis evaluated the effect of standardizing inpatient insomnia medications on trends in physicians’ hypnotic prescriptions for outpatients.

Results

The findings showed a decrease in prescription rates for BZ and non-BZ drugs during the study period. In addition, a decreasing trend was observed for new prescriptions of BZ receptor agonists over the three-month period.

Conclusions

It was suggested that restricting the prescription of BZ for insomnia in inpatient settings could facilitate the appropriate prescription of insomnia medications for outpatients by clinicians.

## Introduction

More than 30% of adults have one of the following insomnia symptoms: difficulty falling asleep, awakening in the middle of the night, early morning awakening, or difficulty sleeping soundly. Insomnia is often accompanied by a variety of psychiatric and physical symptoms. As a result, insomnia causes various human and socioeconomic losses and has become a major public health issue [1]. While benzodiazepines (BZ drugs) are used frequently, they are associated with cognitive decline, staggering due to their muscle relaxant effects, risk of falls, and bone fractures [2-4]. In other countries, the risk of developing dependence from the long-term use of BZ receptor agonists (BZ agonists) has been recognized as a significant concern. Consequently, measures to limit the insurance benefits of BZ agonists have been implemented, such as the exclusion of BZ agonists from insurance benefits in the Netherlands and the imposition of a two-to-four-week prescription limit in the United Kingdom [5-7]. However, in Japan, the promotion of the appropriate use of BZ agonists remains a major challenge. Measures to restrict prescriptions have not been introduced outside psychiatry, and current efforts are limited to issuing reminders to patients.

Guidelines for the use of hypnotic agents in Japan clearly state that BZ drugs should not be used as first-line treatments [1,8]. The Beers Criteria for identifying potentially inappropriate prescriptions, particularly in older adults, advise against the use of BZ drugs [9]. In addition, the STOPP criteria (version 3) recommend limiting the use of BZ drugs to a maximum of four weeks and avoiding their use whenever possible [10,11]. Despite these recommendations, BZ drugs have historically been widely prescribed in Japan. According to UN reports, their usage rates in Japan are higher than in other countries [12]. In addition, in 2019, the Center for Investigation and Support of Medical Accidents issued recommendations for preventing the recurrence of medical accidents. One such recommendation emphasized the careful monitoring of psychotropic drugs, including BZ drugs. Another clearly stated that psychotropic drugs, including BZ-type drugs, should be used with caution [13]. Furthermore, all BZ agonists, including non-BZ drugs, are associated with a risk of amnesia and delirium. New prescriptions for high-risk patients, such as those in the perioperative period and older adults, should be avoided whenever possible [10].

At Hiroshima University Hospital, BZ agonists are indicated for insomnia in the clinical pathway across most departments, and these drugs are kept in some wards. Therefore, in November 2021, with the aim of preventing the initiation of new BZ agonists during hospitalization, the insomnia medication indicator on the ward medication lists and clinical pathways was updated. Patients at risk of delirium were prescribed lemborexant, whereas patients without delirium were prescribed eszopiclone, depending on whether they were at risk of delirium.

Following the revision of insomnia medications in the clinical pathway, BZ drugs are generally avoided for inpatients under this updated pathway. It is also anticipated that physicians will avoid prescribing BZ drugs for insomnia instructions to inpatients not covered by the clinical path by limiting the drugs to those available in hospital wards.

We hypothesized that changes in the drugs kept in the ward and insomnia instructions for the clinical pathway would be effective in motivating physicians to refrain from prescribing BZ agonists. In this study, outpatients’ initial prescription of hypnotic agents before and after the standardization of hypnotic agents for inpatient use was investigated to determine the effect of standardizing inpatient insomnia instructions on the trend of physicians prescribing new hypnotic agents to outpatients.

## Materials and methods

Changes to the clinical path insomnia indicator and the drugs kept in the ward

Aimed at reducing new BZ drug prescriptions during hospitalization, as of November 2021, insomnia medications in the clinical pathway were standardized. The choice between lemborexant and eszopiclone was based on the presence or absence of delirium risk (Table 1). Patients were considered at high risk of delirium if they had at least one of the following risk factors: age ≥70 years, brain disorder, dementia, heavy alcohol use, history of delirium, use of drugs that put the patient at risk of delirium, recent surgery requiring general anesthesia or scheduled for such surgery, and admission to intensive care unit, enhanced care unit, or high care unit [14].

**Table 1:** Clinical pathway insomnia indicator medications

Risk of delirium	Insomnia indicator
Yes	Lemborexant 5 mg once (2.5 mg once if concomitant medications are available); if no effect, add 2.5 mg of lemborexant once
No	Eszopiclone 1 mg once, up to two times

As part of the standardization of the clinical pathways for insomnia, all insomnia medications, other than lemborexant 5 mg and eszopiclone 1 mg, were removed from the list in all wards. On the other hand, since the insomnia indicator in the clinical pathway does not restrict the continuation of brought-in hypnotic agents or new prescriptions of BZ drugs, any BZ agonists the patient had been taking before admission were continued.

Before standardization, the most commonly indicated drugs for insomnia in clinical pathways were brotizolam and ramelteon, with a wide variety of etizolam, rilmazafone, eszopiclone, zopiclone, zolpidem, BZ drugs in 167 pathways, non-BZ drugs in 61 pathways, and melatonin receptor agonists in 26 pathways, for a total of 254 pathways. No orexin receptor antagonists (ORAs) were included. Clinical pathways and drugs kept in wards were revised for 25 departments, excluding pediatrics and hematology, which originally had no clinical pathways or insomnia drugs kept in wards.

Analysis of the prescription rate of hypnotic agents before and after standardization

Period

The analysis period spanned from November 2020 to October 2022. The number of outpatients prescribed hypnotic agents before and after standardization was compared between November 2020 and October 2021 (pre-standardization period) and November 2021 and October 2022 (post-standardization period), following the implementation date of the standardization of drugs indicated for insomnia in the clinical pathway and changes in the number of drugs kept in the wards.

Insomnia Medications

The study included 12 hypnotic agents (nine BZ drugs: triazolam, lormetazepam, rilmazafone, brotizolam, etizolam, flunitrazepam, estazolam, nitrazepam, and quazepam; three non-BZ drugs: zolpidem, zopiclone, and eszopiclone; two ORAs: lemborexant and suvorexant; and melatonin receptor agonist: ramelteon), all listed as principal hypnotic agents in the “Guidelines for the Appropriate Use and Withdrawal of Hypnotic Agents 2014.”

Etizolam was sometimes taken during the day for anxiety purposes, but cases in which it was prescribed for purposes other than insomnia or before going to sleep were excluded.

Analysis Method

We analyzed the hypnotic prescription in outpatients by retrospectively reviewing medical records and used descriptive statistics. The number of patients prescribed the target hypnotic agents, including both regular and abortive use, was obtained from the electronic medical record data warehouse. When multiple targeted hypnotic agents were prescribed, each drug was counted for a single patient. Patients who received multiple prescriptions of hypnotic agents containing the same active ingredient, including both brand-name and generic formulations, during the study period were counted as a single. However, patients in psychiatry, pediatrics, pediatric surgery, and those aged <15 years were excluded due to age or treatment.

Prescription rates were calculated for each period and compared to assess the impact of standardization. The prescription rate was calculated by dividing the number of patients prescribed each hypnotic agent during the study period by the total number of patients prescribed the target hypnotic agents (prescription rate (%) = number of patients prescribed each hypnotic agent/total number of patients prescribed the target hypnotic agents × 100).

To analyze the changes in new and continuing prescriptions, data from August to October 2021 and August to October 2022 were compared. For this study, “New” was defined as patients who had not received a prescription for the hypnotic agent within the past three months.

In addition to examining the impact of changes in outpatient physicians, an analysis was conducted in 2021 and 2022. We compared the data of the list of outpatient physicians as of April of each fiscal year.

Nationwide analysis of the prescription of hypnotic agents

To compare the prescribing trends of BZ between our hospital and other institutions in Japan, data were obtained from the National Database of Health Insurance Claims and Specific Health Checkups of Japan (NDB), an anonymous database of health insurance claims and specific health checkups, to determine the volume of prescriptions of sleep sedatives for outpatients in FY2021 and FY2022, which were close to the analysis period. Since the top 100 drugs in each drug category in FY2021 and the top 300 drugs in each drug category in FY2022 are publicly available, data from the top 100 drugs in both cases were used. The relevant drugs were then extracted from the list of 112 hypnotic sedatives and anxiolytics within the drug category of the drugs used in this study.

Ethics approval

This study was approved by the Ethical Committee for Epidemiology of Hiroshima University (approval E2022-0041).

## Results

Hypnotic agents’ prescription rates

Table 2 shows the prescription rates of hypnotic agents before and after the standardization process. Target hypnotic agents were prescribed to 2,744 and 2,539 outpatients before and after standardization, respectively. Before standardization, 1,654 patients were aged ≥65 years, and 1,090 patients were aged <65 years, with a median age of 69 years (17-98 years). After standardization, 1,529 patients were aged ≥65 years, and 1,010 were aged <65 years, with a median age of 69 years (17-98) (Table 3). The prescription rate of BZ drugs decreased from 36.2% (994/2,744) to 33.5% (851/2,539), whereas that of non-BZ drugs declined from 35.7% (979/2,744) to 31.8% (808/2,539).

**Table 2:** Prescription rate of hypnotic agents before and after the standardization process BZ, benzodiazepines; GABAA, gamma-aminobutyric acid type A; ORA, orexin receptor antagonists

Hypnotic agents			Before standardization (November 2020-October 2021)		After standardization (November 2021-October 2022)	
			Number of patients	Prescription rate (%)	Number of patients	Prescription rate (%)
GABAA receptor (BZ receptor) agonist	BZ	Triazolam	128	4.7	112	4.4
		Lormetazepam	29	1.1	26	1
		Rilmazafone	58	2.1	48	1.9
		Brotizolam	492	17.9	417	16.4
		Etizolam	147	5.4	135	5.3
		Flunitrazepam	85	3.1	66	2.6
		Estazolam	35	1.3	28	1.1
		Nitrazepam	15	0.5	12	0.5
		Quazepam	5	0.2	7	0.3
	Non-BZ	Zolpidem	524	19.1	420	16.5
		Zopiclone	78	2.8	67	2.6
		Eszopiclone	377	13.7	321	12.6
Total of BZ receptor agonist			1,973	71.9	1,659	65.3
ORA		Lemborexant	182	6.6	436	17.2
		Suvorexant	399	14.5	293	11.5
Melatonin receptor agonist		Ramelteon	190	6.9	151	5.9
Total			2,744	100	2,539	100

**Table 3:** Patient background

Age	Before standardization (November 2020-October 2021), N = 2,744	After standardization (November 2021-October 2022), N = 2,539
Mean (range)	69 (17-98)	69 (17-98)
≥65 years	1,654	1,529
<65 years	1,090	1,010

The drugs that showed particularly significant decreases in the number of patients prescribed were brotizolam, which decreased from 17.9% (492/2,744) before standardization to 16.4% (417/2,539) after standardization; flunitrazepam, from 3.1% (85/2,744) to 2.6% (66/2,539); and zolpidem, from 19.1% (524/2,744) to 16.5% (420/2,539). The total prescription rate for BZ and non-BZ drugs decreased from 71.9% (1,973/2,744) to 65.3% (1,659/2,539), whereas the rate of ORA prescriptions increased from 21.2% (581/2,744) to 28.7% (729/2,539).

Changes in the prescription of hypnotic agents to outpatients

Table 4 shows the results of an analysis of new prescriptions for BZ, extracting data from August to October 2021 and 2022. Before and after standardization, 86.4% (1,513/1,752) and 89.2% (1,442/1,616) of outpatients continued their prescriptions, respectively. There were 239 and 174 new prescriptions before and after standardization, respectively. Among newly prescribed hypnotic agents, the combined prescription of BZ and non-BZ medications decreased from 55.2% (132/239 patients) to 51.7% (90/174 patients), whereas the percentage of ORA prescriptions for new patients increased from 38.5% to 43.7%. The three-month total (new + continuation) prescription trends were consistent with the one-year trend (Table 2).

**Table 4:** Rate of new prescriptions, data extracted from August to October 2021 and 2022 BZ, benzodiazepines; GABAA, gamma-aminobutyric acid type A; ORA, orexin receptor antagonists

Hypnotic agents			Before standardization (August to October 2021)				After standardization (August to October 2022)			
			Total		New		Total		New	
			Number of patients	Prescription rate (%)	Number of patients	Prescription rate (%)	Number of patients	Prescription rate (%)	Number of patients	Prescription rate (%)
GABAA receptor (BZ receptor) agonist	BZ	Triazolam	82	4.7	9	3.8	74	4.6	4	2.3
		Lormetazepam	21	1.2	2	0.8	21	1.3	1	0.6
		Rilmazafone	29	1.7	5	2.1	21	1.3	5	2.9
		Brotizolam	335	19.1	22	9.2	292	18.1	15	8.6
		Etizolam	101	5.8	8	3.3	103	6.4	6	3.4
		Flunitrazepam	54	3.1	0	0	48	3	1	0.6
		Estazolam	24	1.4	0	0	21	1.3	0	0
		Nitrazepam	9	0.5	3	1.3	8	0.5	0	0
		Quazepam	4	0.2	0	0	5	0.3	2	1.1
	Non-BZ	Zolpidem	407	23.2	37	15.5	274	17	20	11.5
		Zopiclone	53	3	10	4.2	45	2.8	5	2.9
		Eszopiclone	211	12	36	15.1	189	11.7	31	17.8
Total of BZ receptor agonist			1,330	75.9	132	55.2	1,101	68.1	90	51.7
ORA		Lemborexant	111	6.3	56	23.4	246	15.2	51	29.3
		Suvorexant	216	12.3	36	15.1	173	10.7	25	14.4
Melatonin receptor agonist		Ramelteon	95	5.4	15	6.3	96	5.9	8	4.6
Total of ORA and melatonin receptor agonist			422	24.1	107	44.8	515	31.9	84	48.3
Total			1,752	100	239	100	1,616	100	174	100

Outpatient physicians

Changes in prescribing physicians may affect the content of prescriptions; therefore, changes in outpatient physicians were examined: in FY2021 and FY2022, 30 departments had outpatient physicians, with 402 and 416 physicians in charge, respectively, and 80% of them remained in charge (Table 5).

**Table 5:** Changes in outpatient physicians

Fiscal year	2021	2022
Number of outpatient physicians	402	323 (continuing); 93 (new)

National analysis of prescriptions for hypnotic agents

To ascertain the changes in prescriptions for hypnotic agents over time, the number of prescriptions for hypnotic agents was analyzed using the NDB open data.

The number of prescriptions in FY2021 was 7.87 × 10⁸ for non-BZ drugs and 10.7 × 10⁸ for BZ drugs, for a total of 18.6 × 10⁸ prescriptions for non-BZ and BZ drugs. The total number of prescriptions for ORA and melatonin receptor agonists was 6.70 × 10⁸. The FY2022 prescription count was 7.98 × 10⁸ for non-BZ drugs and 10.3 × 10⁸ for BZ drugs, for a total non-BZ prescription count of 18.3 × 10⁸. The total prescription count for ORA and melatonin receptor agonists was 8.02 × 10⁸ (Table 6).

**Table 6:** Number of prescriptions for hypnotic agents from the national survey BZ, benzodiazepines; ORA, orexin receptor antagonists

Fiscal year	2021	2022
BZ	10.7 × 10⁸	10.3 × 10⁸
Non-BZ	7.87 × 10⁸	7.98 × 10⁸
ORA and melatonin receptor agonist	6.70 × 10⁸	8.02 × 10⁸

In the NDB Open Data, which reflects national medical receipt data, the total number of prescriptions for ORA and melatonin receptor agonists increased by 16.5% from FY2021 to FY2022, whereas the total number of prescriptions for non-BZ and BZ drugs decreased by only 1.7% from FY2021 to FY2022, remaining nearly flat.

## Discussion

To promote the appropriate use of BZ agonists, the standard drugs were set in the clinical pathways for inpatients as lemborexant for those at risk of delirium and eszopiclone for those not at risk of delirium. For patients not covered by the clinical pathway, insomnia medications were also limited to lemborexant and eszopiclone in the ward. Although other hypnotic agents can be prescribed, lemborexant and eszopiclone are primarily used due to their ready availability in the ward [15].

Eszopiclone, a non-BZ drug, was set on the clinical pathway as the standard drug for patients without delirium risk. This decision was based on the recommendation of the Japanese Society of Sleep Research guidelines, which favor eszopiclone as the drug of choice in the absence of delirium risk, while also considering the educational effect of raising awareness among providers about the risk of delirium associated with non-BZ drugs. There were no domestic guidelines on insomnia medications after the launch of ORA in 2021. In addition, it had not been fully used since ORA had been launched for a short period, and there were concerns about carryover due to the long half-life of the drug [1].

Hypothesizing that physicians’ prescription behavior regarding BZ agonists would change as a result of standardizing clinical pathways and drugs kept in the ward, the number of hypnotic agent prescriptions for outpatients before and after standardization was investigated. The results showed a decreasing trend in the percentage of BZ agonist prescriptions after standardization (Table 2, Table 4). Compared to eszopiclone prescriptions on the clinical pathway, zolpidem and brotizolam still have a higher number of prescriptions, but the rate of total prescription reduction is higher (zolpidem is 2.6% and brotizolam is 1.5%) than eszopiclone (1.1%), suggesting a certain reduction effect after the intervention (Table 2). Additionally, there was an increase in the number of patients prescribed lemborexant. However, considering that this was a before-and-after comparison with different prescription periods and the fact that prescribing lemborexant became easier due to the lifting of the administration period restriction in May 2021, it was difficult to statistically analyze the change in prescriptions.

In clinical practice, discontinuing or switching BZ agonists is challenging, partly because of past prescription practices and patients’ long-term dependence [10]. Since withdrawal symptoms may occur in patients with established physical dependence due to abrupt discontinuation, tapering is the basic method of decreasing the dose over time [16], and many patients also experience anxiety about dose reduction. Proper education on sleep hygiene and patients’ decision-making processes must also be adequately addressed. It is crucial to avoid initiating new prescriptions to reduce the use of BZ agonists. This study examined the changes in hypnotic agents limited to new prescriptions and found a decreasing trend in the use of BZ agonists, such as triazolam, brotizolam, and zolpidem. These drugs were incorporated into clinical pathways prior to standardization. These results suggest that improving physicians’ inpatient prescription practices can positively influence outpatient prescription patterns.

The proper use of BZ agonists is a national policy, and in 2017, the BZ agonist package insert was revised to alert the public to the risk of drug dependence, even within the approved dosage range. The Pharmaceuticals and Medical Devices Agency (PMDA) issued a “Request for Proper Drug Use from PMDA” regarding dependence on BZ agonists. In 2024, the PMDA issued another “Request for Appropriate Use of Drugs from PMDA” to address the issue of long-term use of BZ agonists through continuous administration and the resulting physical dependence [17]. This analysis found a decrease in the number of prescriptions for BZ agonists; however, if prescriptions for BZ agonists are decreasing nationwide, this cannot be solely attributed to the standardization of insomnia medications. Therefore, the number of BZ agonists prescribed by physicians nationwide from FY2021 to FY2022 was compared using the NDB open data. Since no significant change in prescription volume was observed, the decrease in prescriptions of BZ agonists at our outpatient clinic is speculated to be due to the standardization of medications kept in the ward and the development of clinical pathways. However, because the NDB database is based on the total prescription volume and not the number of patients, it is possible that the change was not observed because of the high prescription volume of continuing patients.

Given the large turnover of physicians at our hospital, concerns arose about the impact of the changes in prescribing physicians on prescriptions. Therefore, the turnover of outpatient physicians in FY2021 and FY2022 was examined, revealing that 80% of prescribing physicians remained consistent in outpatient settings, suggesting that the impact of changes in prescribing physicians was minimal.

It is not easy to effect a behavioral change in a physician’s long-accustomed prescribing practices. Furthermore, changing sleeping pills is a significant burden for physicians in outpatient settings, where consultation time is limited in acute care hospitals. Under such circumstances, it is significant that the change in prescribing trends among outpatient physicians was observed by operationally guiding them to avoid BZ-based drugs in their inpatient prescriptions. In the future, it is hoped that awareness of the risks of long-term BZ prescribing will deepen among both physicians and patients. The limitations of this study are that we did not investigate outcomes associated with changes in prescriptions for insomnia medications (e.g., number of falls and delirium), and we were unable to obtain details of the comorbidities being treated and the steroid dosage being administered at other clinics because the study subjects were outpatients. Thus, future research should be conducted to clarify whether the insomnia medications prescribed in this study were appropriate and the impact of comorbid diseases and steroid administration on the choice of insomnia medications. In addition, it was reported that the proportion of patients prescribed BZ agonists began to decrease, particularly from about 2015, for both patients aged <75 years and those aged ≥75 years. It was suggested that some guidelines affect the prescriptions of BZ agonists [18]. Therefore, the decrease in BZ prescriptions observed in this study is likely to include a long-term decrease that began before the standardization of insomnia medications.

## Conclusions

This study demonstrated that changing insomnia medications kept in wards and the insomnia indicators in clinical pathways from BZ drugs to ORAs may reduce the prescribing of new BZ drugs in outpatients. A large prospective intervention study is required to evaluate the specific impact of individual physicians’ prescribing behaviors.
